# Elevating sleep to a global health priority: The One Sleep Health framework

**DOI:** 10.1016/j.xcrm.2026.102828

**Published:** 2026-05-22

**Authors:** Masoud Tahmasian, Vincent Küppers, Sarah Genon, Simon B. Eickhoff, Diego A. Golombek, Agustin Ibanez

**Affiliations:** 1 Institute of Neuroscience and Medicine, Brain & Behaviour (INM-7), Research Centre Jülich, Jülich, Germany; 2 Institute of Systems Neuroscience, Medical Faculty and University Hospital Düsseldorf, Heinrich Heine University Düsseldorf, Düsseldorf, Germany; 3 Department of Nuclear Medicine, Faculty of Medicine and University Hospital Cologne, University of Cologne, Köln, Germany; 4 Laboratorio Interdisciplinario del Tiempo (LITERA), Department of Life and Behavioral Sciences, Universidad de San Andrés/CONICET, Buenos Aires, Argentina; 5 Latin American Brain Health Institute (BrainLat), Universidad Adolfo Ibáñez, Santiago, Chile; 6 Global Brain Health Institute, Trinity College Dublin, Dublin, Ireland; 7 Department of Biophysics, School of Medicine, Istanbul Medipol University, Istanbul 34815, Türkiye; 8 Barcelonaβeta Brain Research Center (BBRC), Pasqual Maragall Foundation, 08005 Barcelona, Spain; 9 Cognitive Neuroscience Center (CNC), Universidad de San Andrés, Buenos Aires, Argentina; 10 GIGA-CRC-Human Imaging, University of Liège, Liège, Belgium

**Keywords:** Sleep Health, One Health, Environment, Exposome

## Abstract

Sleep is a non-negotiable necessity for humans and animals and a cornerstone of health. Research is recently undergoing a paradigm shift, moving from a focus on individual factors to macro-level influences on sleep health. The physical, social, and lifestyle exposome increasingly disrupts human and animal sleep, highlighting shared vulnerabilities and potential health risks across species within shared ecosystems. Yet, the interactions among sleep, whole-body health, and exposome factors remain elusive. Here, we propose a novel integrative *One Sleep Health* framework that recognizes sleep as a fundamental pillar of planetary health and highlights how environmental factors influence it in the modern era. It incorporates the *sleep capital* concept—the cumulative health, social, and economic benefits of high sleep quality—into a global sleep health agenda. By adopting a transdisciplinary lens encompassing neuroscience, medicine, environmental science, and public health, this framework aims to bridge critical gaps in global sleep research and diplomacy.

## Teaser

Sleep health is increasingly disrupted by physical, social, and lifestyle factors across species, revealing shared vulnerabilities within ecosystems. Tahmasian et al. introduce the *One Sleep Health* framework, positioning sleep as a pillar of planetary health to guide global research, policy, and public health strategies to mitigate environmental impacts on sleep health.

“Human Beings are parts of one body;

In creation, they are indeed of one nature;

If one member suffers unease;

Other members uneasy will remain;

If you have no sympathy for human pain;

The name of human, you can not pertain.”

(Saadi Shirazi, 1210-1291/1292)

## Background

Healthy sleep is a non-negotiable necessity and a fundamental pillar of overall health for humans and animals.^1^^,^^2^ According to the World Health Organization, health is a state of physical, mental, and social well-being, not merely the absence of disease,^3^ underscoring that optimal sleep contributes to all facets of well-being. Although there is no consensus on a definition of sleep health, it is considered a heterogeneous, multidimensional construct, generally conceptualized in terms of sleep satisfaction, duration, timing, and regularity.^4^ Sleep disorders such as insomnia disorder and obstructive sleep apnea (OSA) are a growing public health concern worldwide and have become one of the most prevalent clinical conditions in modern societies, affecting roughly one-third of the global population.^5^^,^^6^ The prevalence of abnormal sleep durations and insomnia symptoms in a US population study increased annually from 24.44% in 2005–2006 to 30.58% in 2017–2018.^7^ A large-scale, cross-country meta-analysis estimated the overall prevalence of insomnia disorder as 12.4%.^5^ OSA can also affect nearly 1 billion people worldwide, based on literature-based analyses.^6^^,^^8^ Poor sleep health results in annual losses of up to $680 billion across five industrialized countries due to adverse medical and public health outcomes.^9^ Numerous interconnected individual, social, and environmental factors can shape sleep health, underscoring the need for multilevel interventions across families, schools, workplaces, media, and policy to reduce sleep disturbances and their consequences.^4^

Over the last few decades, our environment and societies have undergone significant changes that can negatively impact planetary sleep. Humans and animals are exposed to various internal and external factors (the so-called exposome)^10^^,^^11^,^12^ throughout their lifetimes. The exposome comprises three main domains: 1) the *physical exposome* encompasses rising global temperatures, air, noise, and light pollution, artificial lighting, heavy traffic, and the shrinking of green urban areas; 2) the *social exposome* encompasses a range of factors, including socioeconomic disparities, elevated family and occupational stress, circadian dysrhythmia due to misalignment between endogenous and exogenous timing (e.g., social jet lag—discrepancies between preferred and imposed sleep patterns on weekdays and weekends), and shift work; finally, 3) the *lifestyle exposome* include unhealthy habits such as poor diet, low physical activity, and higher consumption of tobacco, alcohol, and caffeine, as well as excessive social media use, digital overload, and prolonged screen exposure. The escalating exposome challenges have a cumulative and devastating effect across the lifespan, leading to accelerated brain aging,^10^^,^^11^,^13^ mental health problems,^14^ and sleep disturbances worldwide.^15^^,^^16^

Despite this growing recognition, sleep health remains underprioritized in many national and international health strategies, especially in low-resource settings, which often face the most significant challenges. Moreover, there is a limited understanding of the interplay among sleep health, brain and body health, and exposome factors. The lack of global sleep hygiene education and the insufficient integration of sleep as a priority in public health policies have hindered coordinated international action on sleep health worldwide. Addressing these gaps requires a more synergistic, multimodal, and integrative approach that spans the various dimensions influencing global sleep health.

**Figure 1:**
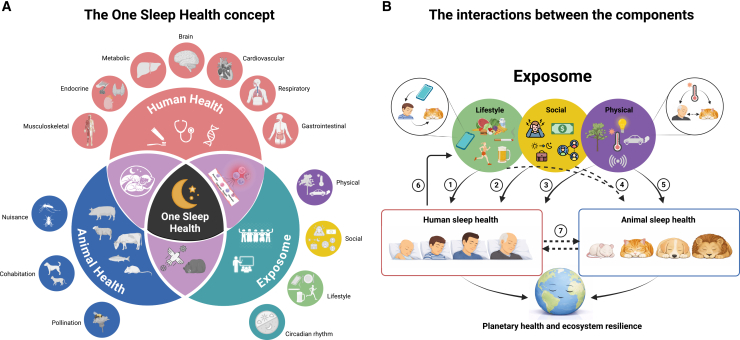
The structure of the One Sleep Health framework (A) This model represents a complex interaction between the human brain and body, animal health, and exposome factors. Sleep has bidirectional relationships with brain structure and function, as well as with various organ systems, including the cardiovascular, metabolic, respiratory, gastrointestinal, musculoskeletal, and endocrine systems. The roles of physical and climatic changes, social factors, and lifestyle choices in shaping humans’ sleep patterns across diverse cultural, socioeconomic, and educational groups have been repeatedly reported.^15^^,^^16^ Exposome factors, including light and noise pollution, can affect the human circadian rhythm, and such poor sleep can lead to inflammation across multiple body systems. Environmental changes may also influence animals’ sleep patterns, their daytime activities, migration or hibernation in some species, and even pollination behaviors. Human and animal sleep health are interrelated; for example, flies and mosquitoes can disrupt human sleep, especially in rural or peri-urban environments. (B) The interaction between the components of the One Sleep Health model. Lifestyle choices and social factors can affect sleep health (nos. 1 and 2) and may indirectly influence animal sleep (no. 4), although their influence depends on factors such as age, sex, and work or home location. For example, social media is more commonly used by children, adolescents, and young adults than by older adults. Moreover, work-related stress may affect sleep patterns of young and middle-aged adults. Men and women experience different types of work- or family-related stress and social support and may have different lifestyles, all of which can affect their sleep behaviors. The lifestyle and sleeping habits of pet owners (e.g., delayed phase, insomnia) can indirectly impact the sleep health of domestic animals (no. 4). Environmental pollution (e.g., light, noise, and air pollution) and climate change (high temperature) can alter sleep patterns in both humans (especially older adults) and animals (no. 3 and 5). These disturbances may induce inflammation and maladaptive physiological responses, negatively affecting overall health and well-being. Sleep disturbances can exacerbate environmental degradation by affecting individual mood and social interactions in family, school, and work environments, and by promoting further unhealthy lifestyle behaviors (e.g., increased alcohol consumption), creating a complex bidirectional feedback loop between human health and the environmental exposome (no. 6). Urbanization affects domestic and wild animals by altering their natural habitats and behaviors (e.g., through increased light and noise pollution and later sleep timing). This can lead to increased stress, increased sympathetic activity overnight, reduced deep sleep, and potential health consequences in animals, which, in turn, can affect human sleep during cohabitation and, in turn, human health. Similarly, in shared living environments, sleep disturbances in humans can lead to poor sleep in domestic pets (no. 7). Thus, optimal sleep health across species is essential for planetary health and ecosystem resilience. *Dash lines reflect areas with less existing evidence*. *This figure was created in Biorender* (https://BioRender.com/y8aqiyn).

While sleep research has traditionally focused on individual contributing factors, there is now a realization that macro-level forces—from climate change to urbanization and wildlife ecology—may profoundly influence sleep health in humans and animals. Thus, we propose to bridge these gaps by promoting an integrative *One Sleep Health* (OSH) model that, for the first time, recognizes the transdisciplinary, integrative link among human sleep, animal sleep, and the physical, social, and lifestyle exposome (Figure 1A). The *One Health* model emerged approximately two decades ago as a sustainable approach that integrates human health with the impact of domestic and wild animals, plants, and ecosystems.^17^^,^^18^ In this model, researchers, clinicians, government officials, and policymakers at regional and global levels develop shared databases, identify innovative recommendations for emerging diseases, and guide the development of long-term global action plans for health threats. Although not free of challenges (Box 1), the OSH approach is timely and novel in addressing today’s pressing exposome changes in sleep health. Moreover, two recently proposed concepts make a significant advance in the OSH framework: 1) *sleep capital*,^19^ defined as the compound social, economic, and health benefits derived from adequate sleep parameters, resulting in increased individual and social well-being; and 2) s*leep diplomacy*,^20^ characterized by local and global sleep-promoting interventions and strategies. These concepts contribute to the OSH framework by providing strategies for sharing information among different stakeholders (see Box S1).

Box 1 **Key challenges for an optimal One Sleep Health agenda**

**Sleep health is shaped by a convergence of physical, social, and lifestyle factors that are increasingly misaligned with our biological needs**

**Climate change and extreme heat**

Global warming reduces sleep duration and quality, with projections indicating a loss of 50–58 h of sleep annually per person by the end of the century, especially among older adults, women, and those in hotter, low-income regions. Climate-related stressors, such as heatwaves and displacement events, also disrupt sleep in humans and animals.

**Environmental pollution**

Light, noise, and air pollution collectively impair sleep by disrupting circadian rhythms, increasing nighttime arousals, and aggravating respiratory issues. Artificial lighting suppresses melatonin production and delays sleep onset. Noise pollution contributes to fragmented sleep and elevated cardiovascular risk, while air pollution is linked to insomnia and OSA. Urban planning strategies prioritizing dark skies, clean air, and quiet nights can protect human and animal sleep.

**Digital overload**

Excessive screen time, especially in the evening and overnight, delays circadian timing and reduces sleep. Blue-light exposure and constant digital engagement contribute to digital insomnia, particularly among youth. Managing this challenge requires individual behavior change, guidance from schools and workplaces, and public campaigns promoting digital curfews.

**Social stressors and 24/7 culture**

Economic hardship and disparity, long work hours, shift work, social jet lag, and a hyper-connected society elevate stress and promote irregular sleep schedules. Marginalized populations often face compounded barriers to restorative sleep. Cultural shifts and policy reforms, such as flexible work arrangements, protected rest periods, and equitable housing, are essential.

**Unhealthy lifestyles**

Poor diet, sedentariness, and substance use all impair sleep quality. Caffeine, alcohol, nicotine, and large meals late at night disrupt sleep cycles. Public health efforts must link sleep hygiene and behavioral change interventions, including nudge design, cognitive-behavioral therapy, and mindfulness.

**Inter-individual variability**

Sleep needs vary across individuals, cultures, and geographic locations, yet the definition of sleep health often overlooks these dynamics.

**Recommendations for developing sleep health as a global priority**

**Make sleep a public health priority**

**Addressees: Governments and global health bodies (e.g., WHO, UN).** Governments and global health bodies (e.g., WHO, UN) should officially recognize sleep as essential to health. Sleep indicators must be included in health surveillance and policy targets, such as reducing the prevalence of short sleep or limiting the night-shift burden. A global task force could coordinate actions and share best practices. All policies must include vulnerable groups, such as shift workers, older adults, patients with circadian rhythm disruptions, and low-income populations.

**Promote sleep awareness**

**Addressees: Public health agencies, environmental authorities, and sustainability stakeholders.** Raising international awareness and implementing sleep-related behavioral and environmental interventions through the OSH approach can enhance human and animal health, well-being, and resilience. This strategy aims to enhance human and animal health and support sustainable development. By focusing on sleep in relation to environmental and societal factors, the OSH approach facilitates the identification of subtle or hidden influences on global sleep health. This, in turn, supports the development of sustainable societies and ecosystems by enabling the identification and adaptation of subtle or hidden environmental and social factors that influence sleep worldwide.

**Promote sleep hygiene education**

**Addressees: Education systems, workplaces, healthcare providers, and the public.** Public campaigns should raise awareness about the benefits of sleep hygiene and how to improve sleep health through consistent schedules, limited screen use, and better bedtime routines. Schools should teach sleep science. Workplaces can support better sleep by limiting after-hours communication and encouraging healthy routines. Healthcare providers should ask about sleep in routine visits. Clear, science-based messaging can help change cultural norms and reduce stigma surrounding the importance of prioritizing sleep.

**Train clinicians and animal health experts**

**Addressees: Medical, veterinary, and wildlife training institutions and professional bodies.** Medical and veterinary curricula must include *One Sleep Health*. Doctors, nurses, and psychologists need training in sleep physiology and in disorders such as insomnia and OSA. Veterinarians should understand how lighting and stress affect animal sleep. Wildlife experts should consider sleep in welfare evaluations.

**Implement sleep diplomacy policies**

**Addressees: Policymakers, urban planners, education authorities, and labor regulators.** Countries should regulate light, air, and noise pollution and design circadian-friendly environments. Schools can delay start times for adolescents. Workplaces should limit excessive overtime and night shifts. National policies can support digital curfews and safer shift-work guidelines. Future global sleep health indices could monitor progress. Policies such as phasing out daylight saving time or incorporating sleep recovery into disaster aid plans are simple yet impactful.

**Boost research and global surveillance**

**Addressees: Funders, researchers, technology developers, and global surveillance bodies.** Invest in large-scale, diverse, and longitudinal studies. Use (neuro)biological data, wearable technology, and smartphone data (with privacy safeguards) to map sleep trends. Champion interdisciplinary research linking sleep to exposomes and health. Support sleep technology innovation and create global sleep data repositories.

Framing sleep within the *One Health* approach requires synergistic and syndemic models that integrate interactions among the brain, body, and the environment. These factors do not act in isolation. A polluted, noisy urban environment often coexists with high psychosocial stress, socioeconomic disparities, and lifestyle challenges, jointly degrading sleep health. Efforts to improve environmental conditions and ecosystem resilience (cleaner air, greener cities, climate change mitigation) often coincide with enhancing social well-being and socioeconomic status (reducing stress, reducing inequity, fostering community resilience). The OSH model provides a holistic, interdisciplinary operational framework for understanding these multi-level interactions. Figure 1B illustrates the structure of the OSH model, describing its domains, hypothesized relationships, and potential feedback links. The model consists of three primary domains, including human sleep health, animal sleep health, and the exposome, which comprises lifestyle, physical, and social factors. The exposome approach offers a promising framework to quantify how cumulative environmental burdens translate into sleep loss and subsequent health outcomes in the brain and body. Large-scale epidemiological projects linking human health records with environmental datasets (e.g., pollution, noise, climate indices, and social factors) are beginning to tease out causal pathways by which physical and social exposome factors influence sleep health. Ultimately, the insights gained must inform policy. Recognizing that sleep is a critical aspect of public health, influenced by and impacting our environment, represents a paradigm shift that will drive more effective solutions to today’s sleep-loss epidemic. The integrated OSH model, spanning neuroscience, internal medicine, psychology, epidemiology, environmental science, and veterinary science, may help address these challenges. In the following sections, we review the existing evidence for all these domains and their interactions.

## The need for a global perspective in sleep health

Sleep need is universal but dynamic and flexible across individuals, requiring tailored assessments and ecologically sensitive approaches.^21^ Subjective sleep duration reports from 63 countries showed that East Asians reported shorter sleep durations than Europeans or Americans.^22^ A study of ∼220,000 users of wearable sleep-tracking devices across 35 countries confirmed that East Asians had shorter sleep durations, later sleep timing (delayed sleep phase), different social jet lag patterns, and lower sleep efficiency than people in Western Europe, North America, and Oceania.^23^ South American, Middle Eastern, and East Asian cultures tend to have delayed (more evening) chronotypes and daytime napping.^23^ These differences may be explained by geographic and latitude, varying light and heat exposure, economic inequity, and, more importantly, cultural differences in prioritizing sleep over work or late-evening social activities. Notably, prevailing sleep hygiene recommendations mainly reflect Western habits and do not incorporate cultural variations in sleep-wake patterns (e.g., monophasic vs. biphasic sleep [siesta]) or disparities in sleep efficiency across regions, limiting their global applicability.^23^ Thus, we believe that the definition of *normal sleep* varies across cultures and regions and should not be limited to standard criteria based solely on Western sleep habits. Given the unique brain-body-environment interactions in humans and animals across countries, the variability in sleep patterns underscores the need for a global perspective to identify and adapt to the specific environmental and social factors that influence sleep health worldwide.

## Individual biological susceptibility

Individuals (human or non-human animals) differ in how they respond to environmental influences. The 3P model of insomnia disorder, including predisposing, precipitating, and perpetuating factors, offers a valuable framework for understanding how biological, psychological, and socio-environmental elements contribute to the onset, maintenance, and treatment of clinical conditions.^24^^,^^25^ *Predisposing factors* are long-standing characteristics that exist for years before the onset of any sleep disorder. These include genetic vulnerability, sex-specific risk (e.g., a higher likelihood of insomnia in females and OSA in males), normal or accelerated aging, family history, and specific personality traits (e.g., obsessive-compulsive personality traits in insomnia).^26^ Genetic variation plays a substantial role in shaping individual sleep patterns and the risk of developing sleep disorders. An exome-wide study in 450,000 individuals identified specific genetic variants that influence sleep duration, insomnia symptoms, daytime sleepiness, chronotype, and snoring, and one of these genes (ANKRD12) was associated with cognition and inflammatory traits.^27^ A multivariate genome-wide association study (GWAS) demonstrated that sleep health comprises several interrelated yet genetically distinct domains, characterized by genomic enrichment in brain regions such as the cerebellum and basal ganglia.^28^ Interestingly, a large-scale gene-by-environment interaction study found that individuals carrying specific genetic variants exhibit a more pronounced shift toward a morning chronotype in response to daylight exposure.^29^ These findings suggest that genetic predispositions can shape sleep health phenotypes and modulate the extent to which environmental exposures can impact individual variability in sleep-wake behavior.

The *precipitating factors* of sleep disorders include single, multiple, or recurring events, including environmental or psychosocial stressors (e.g., family- or work-related stress, shift work, job loss, or bereavement) and chronic use of medications that disrupt sleep patterns. While acute triggers often resolve naturally over time, some individuals develop maladaptive coping strategies—known as *perpetuating factors*—that can reinforce sleep disturbances through classical conditioning (e.g., sleep-related negative preoccupation, dysfunctional beliefs about sleep, and excessive rumination), making sleep disorders persistent and difficult to treat.^24^^,^^26^

## Sleep and whole-body: The influence of poor sleep on brain and body health

**Figure 2:**
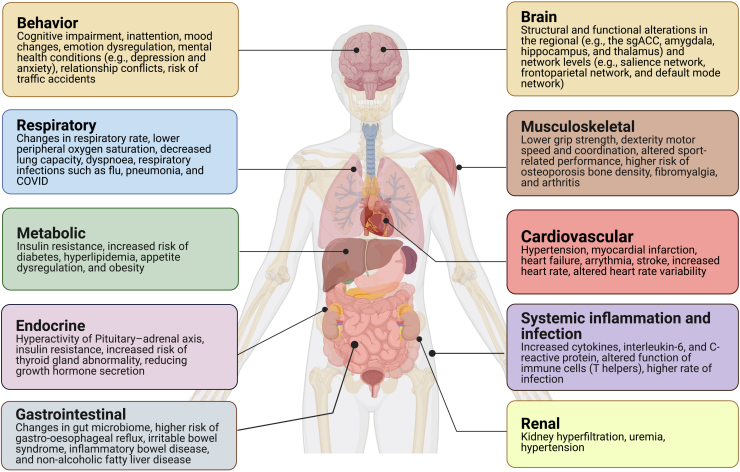
The impact of poor sleep health on the whole body The bidirectional association between poor sleep health and clinical conditions across various body systems, including brain structure and function, behavioral maladaptation, cardiovascular, metabolic, endocrine, respiratory, gastrointestinal, musculoskeletal, and renal system diseases, as well as systemic inflammation and infection.^32^^,^^40^^,^^41^^,^^42^^,^^43^*The figure was created in* Biorender (https://BioRender.com/1i0bl9y).

The neurobiological substrates of sleep are complex. Healthy sleep plays a neuroprotective role in brain aging and cognition.^30^ Large-scale, population-based studies, primarily using the UK Biobank (UKB), have found that around 7 h of sleep is optimal for cognitive performance, with both shorter and longer sleep durations associated with worse physical and mental outcomes.^31^^,^^32^ Similarly, individuals who consistently slept 7 h performed best on a spatial navigation task across various age ranges in 63 countries.^22^ In addition, sleep facilitates the brain’s glymphatic system, which clears metabolic waste products (e.g., β-amyloid proteins) from brain tissue (see reviews^33^^,^^34^). Animal models have demonstrated that this clearance is primarily active during slow-wave sleep via norepinephrine-mediated slow vasomotion,^35^ which likely explains why even a single night of total sleep deprivation can precipitate waste accumulation in the human brain.^36^ Although sleep deprivation and circadian dysfunction promote the accumulation of β-amyloid and tau in the brain,^37^ β-amyloid accumulation also disrupts slow-wave activity and circadian rhythm in older adults, suggesting a bidirectional link between poor sleep and cognitive impairment.^31^^,^^34^ Neuroimaging evidence shows that OSA and insomnia are associated with functional and structural brain alterations, which are also related to cognitive decline and dementia^38^^,^^39^ (Figure 2). Our recent large-scale meta-analysis identified convergent brain abnormalities in the subgenual anterior cingulate cortex and the right amygdala/hippocampus across various chronic sleep disorders, as well as dysfunction of the right thalamus following short-term sleep deprivation.^40^ In summary, the brain needs regular, sufficient, high-quality sleep to maintain mood and cognition, support optimal clearance of neurotoxic waste, and preserve long-term brain integrity, thereby reducing the risk of brain dysfunction and atrophy.

Sleep exerts a pervasive influence on behavior and mental health. Chronic sleep disturbances and insomnia are associated with impaired attention, emotional dysregulation, motor performance, and an increased risk of psychiatric conditions such as anxiety and depression in adolescents, middle-aged, or older adults.^44^^,^^45^^,^^46^ For example, sleep disturbances are the leading predictor of high-risk mental health conditions in adolescents, exceeding the impact of both childhood adversity and family history of affective disorders.^47^ Similarly, poor sleep quality can predict the severity of depressive symptoms in young and middle-aged adults across different age categories.^48^ A large-scale study found that among various lifestyle factors, adequate sleep provides the greatest reduction in depression risk (by 22%), including for single depressive episodes and treatment-resistant depression.^49^ Interestingly, a meta-analysis across 65 randomized controlled trials showed that treating sleep problems can improve mental health.^50^ However, a recent multivariate analysis showed that cognitive psychotherapy for depression cannot reduce insomnia symptoms per se, and such residual insomnia symptoms increase vulnerability to relapse or recurrence of depression.^51^ This evidence underscores the need for a comprehensive approach to managing mental health disorders, with particular attention to interventions that directly target sleep. Altogether, these findings underscore sleep health as a fundamental, modifiable determinant of behavioral and mental health.

Poor sleep health can also adversely affect various body systems, increasing the risk of cardiovascular, metabolic, endocrine, respiratory, gastrointestinal, musculoskeletal, and renal diseases, as well as systemic inflammation, infection, and premature mortality^41^^,^^42^^,^^52^ (Figure 2). During sleep, the body’s organs perform critical restorative functions. When sleep is curtailed or fragmented, both immediate and long-term physiological consequences occur, including activation of the stress response, increased blood pressure, and impaired glucose metabolism. Sleep disturbances and insomnia initiate a cycle of stress responses through a hyperarousal state, triggering the release of stress hormones (including cortisol and adrenaline) and adversely affecting whole-body health.^26^ A review highlighted sleep loss as a risk factor for metabolic disorders that disrupt energy homeostasis and metabolite balance.^43^ Short sleep duration (<6 h) and low sleep quality are linked to a higher incidence of hypertension, coronary artery disease, stroke, obesity, type 2 diabetes, and impaired immune responses.^43^^,^^53^ A recent study based on 448 sleep parameters collected from 16,812 nights of home sleep monitoring in 6,366 adults demonstrated that OSA symptoms can predict insulin resistance, triglyceride levels, and cardiovascular measurements.^41^ Not only do sleep disturbances increase the risk of medical diseases, but certain conditions also modulate sleep patterns in animal models. After a myocardial infarction, the body augments sleep via brain-immune signals, as the injured heart signals the brain to promote deep sleep, which in turn helps limit inflammation and tissue damage.^54^ This cardiogenic sleep-regulation pathway suggests that sleep is integral to the body’s healing response to injury. Unhealthy sleep also undermines the immune system’s effectiveness, e.g., dampening vaccine responses and increasing susceptibility to infections (see review^55^). Over the long run, chronic sleep disorders contribute to systemic inflammation, hormonal imbalances, and autonomic dysregulation, creating a cascade of adverse outcomes, from accelerated arterial plaque build-up to impaired insulin sensitivity and metabolic syndrome.^43^^,^^55^ Existing reviews emphasized that circadian rhythms play a crucial role in regulating energy metabolism.^56^^,^^57^ Circadian disruption due to modern 24/7 demands and lifestyle choices (see below) disrupts sleep/wake schedules and biological clocks, leading to various health issues, including obesity, cardiometabolic syndrome, and cancer.^57^ Understanding the mechanisms that regulate sleep and circadian rhythms may provide insights into behavioral interventions that could lower disease and mortality risk.

These multisystem impacts, from brain to behavior to body health, help explain why habitual poor sleep is associated with higher all-cause mortality.^42^^,^^52^ Extremely irregular sleep timing is a stronger predictor of mortality than the average amount of sleep in the UKB data.^42^ Encouragingly, improving sleep can yield broad health benefits. In general, preserving healthy sleep is as critical for brain, mind, and body health, as it maintains the neural and physiological balance upon which both daily functioning and long-term health depend (Figure 2).

## Sleep and circadian health in the biosphere

Sleep and circadian rhythms, fundamental to maintaining physiological homeostasis, both influence and are shaped by ecosystems, biodiversity, and overall well-being.^2^^,^^57^ Understanding these interactions requires an integrative research perspective that considers biological rhythms across species and environments. In animal health, circadian rhythms play a vital role in regulating sleep, feeding, reproduction, and immune function across species. Artificial light pollution, climate change, and habitat destruction profoundly alter the natural biological rhythms of both domestic and wild animals. Domestic pets are becoming an essential part of many families. Evidence suggests that their circadian rhythms can be affected by artificial light schedules^58^ and can also influence human sleep during co-sleeping,^59^ highlighting the interaction between human and animal sleep.

Environmental changes profoundly alter the natural biological rhythms of both domestic and wild animals. These disruptions affect migration patterns, reproductive cycles, and even disease susceptibility. In the realm of animal health, circadian rhythms play a vital role in regulating sleep, feeding, reproduction, and maintaining optimal immune function across species. These natural clocks interact with human sleep and circadian rhythms in a bidirectional relationship.^60^ Optimizing treatment schedules and animal care based on chronobiological principles in veterinary medicine could enhance health outcomes in pets^61^ and livestock,^62^ whose circadian rhythms also affect rural life and, in turn, human sleep patterns. The microbiome-gut-brain axis directly connects the biosphere and circadian rhythms.^63^^,^^64^ Gut bacterial clocks interact with human biological clocks and are affected by the environment, including feeding, pollution, and pharmacological interactions.^65^ Feeding rhythms drive diurnal oscillations in the intestinal microbiota of both mice and humans. Timed food intake produces a distinct, time-specific microbial profile,^66^ indicating that sleep timing disruptions such as shift work or jet lag lead to aberrant fluctuations and dysbiosis (i.e., an imbalance or disruption in the composition of the gut microbiome, often linked to disease or poor health). In addition, planetary changes (e.g., climate change) adversely affect circadian rhythms and the lifestyles of species involved in pollination or the food chain, thereby indirectly influencing human health and well-being.

The nuisance of flies, mosquitoes, and animal vocalizations (e.g., barking dogs, rooster crowing, livestock, and wildlife sounds) can cause sleep fragmentation, especially in rural environments. Parasites and insects (e.g., pinworms, scabies, lice, bedbugs, and fleas) may cause nocturnal itching and awakening. Sleeping with domestic pets, especially dogs, also increases the risk of awakening during sleep,^67^ leading to poor sleep quality in both species. Nevertheless, the interaction between human sleep health and the animal world remains an area requiring further research. While there is substantial evidence on how human activities impact animal sleep, the reverse link is less clear in the literature. We suggest future studies should focus on the following strategic lines: a) *behavioral synchronization*, investigating how shared living environments influence sleep patterns and circadian rhythms across species; b) *cross-species stress and health impacts*, to understand how social and life stress and poor health in animals relate to their sleep, and how this might affect human caregivers. Thus, optimal sleep health across species is a key pillar of planetary health and ecosystem resilience.

## Environmental exposure and sleep health interactions

**Figure 3:**
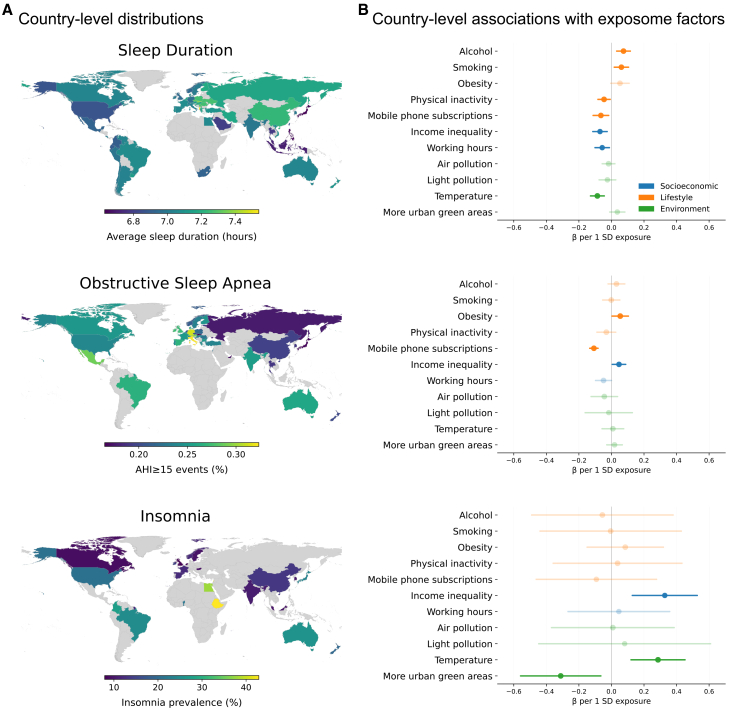
Global distributions of sleep health indicators and their associations with lifestyle, environmental, and socioeconomic exposome factors (A) Country-level distributions (absolute values) of sleep health-related factors. Mean self-reported sleep duration (hours), assessed through the Sea Hero Quest game (2016–2017);^22^ estimated prevalence of obstructive sleep apnea (OSA; Apnoea-Hypopnoea Index ≥15 events per hour), derived from a population-based study using an under-mattress OSA sensor;^69^ and insomnia prevalence (%), derived from reported rates of either insomnia disorder or insomnia symptoms in 2025 meta-analysis.^5^ Gray areas indicate missing or unavailable data. (B) Country-level linear regressions between sleep health-related factors and lifestyle and behavioral, social, and environmental exposures: Points and horizontal lines show regression coefficients (β) and 95% confidence interval (CI) from linear regression models, scaled per one standard deviation increase in each exposure. Exposures include estimated alcohol consumption in 2020 (liters per capita per year) (World Health Organization [via World Bank] [2025] – processed by Our World in Data, https://ourworldindata.org/grapher/total-alcohol-consumption-per-capita-litres-of-pure-alcohol); estimated smoking prevalence among persons aged ≥15 (2022, %) (World Health Organization - Global Health Observatory [2024] – processed by Our World in Data, https://ourworldindata.org/grapher/share-of-adults-who-smoke); estimated age-standardized physical inactivity among adults (2024 meta-analysis, %);^70^ estimated obesity prevalence among adults (2016, BMI ≥ 30, %) (World Health Organization - Global Health Observatory [2024] – processed by Our World in Data, https://ourworldindata.org/grapher/share-of-adults-defined-as-obese); estimated average annual working hours per worker (2019) (Feenstra et al. [2015], Penn World Table [2021] – with major processing by Our World in Data, https://ourworldindata.org/grapher/annual-working-hours-vs-gdp-per-capita-pwt); mobile phone subscriptions (2023, per capita) (International Telecommunication Union [ITU], via World Bank [2025] – processed by Our World in Data, https://archive.ourworldindata.org/20260112-111535/grapher/mobile-cellular-subscriptions-per-100-people.html); income inequality (2018–2023, Gini coefficient) (World Bank Poverty and Inequality Platform [2024] – with major processing by Our World in Data, https://ourworldindata.org/grapher/economic-inequality-gini-index); air pollution (2022, mean PM2.5 concentration in μg/m^3^) (Global Burden of Disease Study 2021 [GBD 2021] Air Pollution Exposure Estimates, IHME, via World Bank [2025] – processed by Our World in Data, https://archive.ourworldindata.org/20260112-111535/grapher/average-exposure-pm25-pollution.html); artificial light pollution (expressed as radiance per 1,000 population based on VIIRS country data from Jurij Stare, www.lightpollutionmap.info, NASA’s Black Marble/Nighttime Lights);^71^ annual average temperature (2019–2024, °C) (https://cds.climate.copernicus.eu/datasets/derived-era5-land-daily-statistics?tab=overview);^72^ and urban green space (2020, % coverage in cities) (https://data.unhabitat.org/pages/open-spaces-and-green-areas). Paler estimates indicate associations that are not statistically significant (*p* ≥ 0.05). All associations reflect ecological co-variation at the country level and do not imply individual-level relationships or causal effects.

Environmental and climate-related changes, including global warming, air, noise, and light pollution, artificial lighting, heavy traffic, reduced green spaces, extreme weather events, and daylight saving time, have been linked to sleep disturbances (see reviews^15^^,^^16^). Comparative chronobiology studies examining sleep across species and habitats (urban vs. wild) can reveal how the environment modulates the general principles of sleep regulation. For example, comparing two indigenous hunter-gatherer communities in the Argentinean Chaco with or without access to electric light reveals essential differences in sleep duration and phase.^68^ Additionally, a combination of social factors and unhealthy lifestyle behaviors is a substantial risk factor for chronic sleep disorders. Global distributions of sleep health factors vary across countries, according to the literature. To characterize how this variation relates to exposome factors, we performed separate country-level linear regression models for each social, lifestyle, and environmental factor and each sleep health indicator (sleep duration, OSA prevalence, and insomnia prevalence). Statistical inference was computed using heteroskedasticity-robust standard errors. These analyses revealed heterogeneous patterns of association between the exposome and different sleep health factors (Figure 3). This section discusses how these interconnected factors influence sleep health.

### Disrupting nature’s cycles and physical environmental factors

Epidemiologic studies have shown that light at night (LAN) exposure is a pervasive form of pollution linked to health issues in humans and wildlife.^73^ Outdoor artificial LAN has been correlated with sleep disorders, obesity, reduced immunity, endocrine dysfunction, cancer, cardiovascular diseases, and mental health problems.^74^ Indoor light intensity also contributes to these conditions in cross-sectional analyses.^75^ Animal studies have shown that constant illumination accelerates aging, enhances spontaneous tumorigenesis, and shortens lifespan.^76^ Melatonin supplementation can prevent the adverse effects of LAN in rodents, suggesting its potential role in cancer prevention in humans.^77^ Exposure to bright artificial LAN suppresses melatonin secretion, increases sleep onset latency, and enhances nocturnal alertness in humans and wildlife.^76^^,^^77^ Ambient conditions shape the monophasic sleep patterns seen in nocturnal primates.^78^ Environmental noise can cause fragmented sleep and insomnia symptoms,^79^ leading to increased heart rate and blood pressure.^80^ The effects of artificial LAN on wildlife, however, require more ecological and chronobiological evidence.^73^

Climate change also poses significant threats to sleep patterns. Rising temperatures and extreme weather have diminished sleep duration and quality, particularly among older adults, low-income groups, and wildlife.^69^^,^^81^^,^^82^^,^^83^ Indeed, animals typically rely on light cues to regulate seasonal physiological processes. Climate change may alter these light-dependent mechanisms, affecting species’ fitness and survival. Global warming may disrupt the balance between internal circadian rhythms and external environmental cues. While humans can adapt to various climates, the rapid pace of climate change presents new challenges. A global study found that warmer nights curtail sleep, with 50–58 h of annual sleep loss per person projected by the end of this century.^82^ Environmental factors can affect gene expression, with sleep restriction leading to substantial reductions in circadian transcripts,^84^ influencing metabolism, immune function, and stress responses. A systematic review synthesizing evidence from 204 studies demonstrated that exposure to chemical pollutants (air pollution, metals, pesticides, solvents, and endocrine-disrupting chemicals) is consistently associated with poor sleep quality, abnormal sleep duration, insomnia, and OSA.^85^ Air pollution also affects glymphatic brain clearance, circadian health, and the risk of dementia (see review^86^). The destruction of natural habitats disrupts the sleep and activity patterns of countless species, resulting in cascading ecological consequences. Such perturbations are reshaping the natural light-dark and thermal cycles that have governed sleep for millennia. Thus, climate change-induced alterations in sleep patterns could have far-reaching consequences for both human and animal sleep health and ecosystems.

### Social exposome and lifestyle contributors to sleep health

Besides physical exposures, the social exposome (socioeconomic and lifestyle factors) also influences our sleep. Modern society’s demands—a 24/7 work culture, shift work, around-the-clock services, and ubiquitous digital connectivity—have led to chronobiological strain. Nocturnal shift work increases the risk of obesity, diabetes, cardiovascular disease, brain abnormalities, mental health problems, and cancer.^87^ Even among non-shift workers, the pressures of round-the-clock lifestyles lead to social jet lag, where people stay up late on weekends or work-free days and struggle to readjust their internal clocks on working days. Big data analyses have revealed that social jet lag is linked to metabolic dysfunction and mood disturbances, including those associated with travel-related jet lag, particularly among adolescents and young adults.^23^^,^^47^

Socioeconomic stressors, inequity, and disparity often induce a heavier burden of sleep problems due to high stress, multiple jobs or long shifts, crowded living conditions, traffic or airport proximity, and limited access to health care. Over 50% of the variation in individuals’ sleep duration and quality can be attributed to societal factors, including working hours, family dynamics, and cultural norms, rather than individual biology.^21^^,^^88^ As mentioned earlier in the 3P model, social stressors (job insecurity, academic pressures on students, pandemics, or regional conflicts) and psychological factors (e.g., chronic stress and comorbid anxiety and depression) can precipitate acute insomnia and, if sustained, chronic sleep disorders.^24^^,^^25^ On the other hand, social support and resilience are associated with a lower rate of sleep disturbances among community-dwelling adults.^89^ Sleep disturbances during global pandemic (e.g., coronavirus disease 2019 [COVID-19]) were quite prevalent and associated with psychological distress, lack of social support, and mental health conditions.^90^

Digital pollution and technology overload is a new challenge to human sleep health. The rise of smartphones, social media, and streaming services means many people extend their waking activities into the night. Excessive screen time displaces sleep opportunity and delays melatonin release. Electronic media use is associated with lower sleep quality and related disturbances.^91^ Problematic late-night use of the internet, gaming, or social media is associated with insomnia symptoms and daytime fatigue. Consistently, a meta-analysis of longitudinal studies reported that prolonged or dysfunctional digital media use is linked to subsequent poor sleep health and increased risk of insomnia.^92^ This may have adverse impacts on adiposity, blood lipids, and glycemic control, as well as emotional outcomes, particularly in adolescents and young adults.^93^ The National Sleep Foundation consensus summarized three points regarding the link between sleep and digital technology: 1) overall, children’s and adolescents’ sleep health is negatively affected by screen use; 2) engaging with screen-based content before bedtime further disrupts subsequent sleep quality; 3) specific behavioral interventions can help reduce the harmful impact of screen use on sleep.^94^

Unhealthy personal habits also feed into this vicious cycle. For instance, late caffeine consumption disrupts sleep via adenosine receptors.^95^ While alcohol can induce sleep onset in some individuals, it causes sleep fragmentation and disrupts REM sleep.^96^ Cigarette smoking is also associated with insomnia and reduced sleep durations.^97^ Similarly, physical inactivity and sedentary lifestyles can exacerbate sleep difficulties.^98^ Consuming high-sugar, high-calorie meals—especially late at night—can disrupt the sleep-wake cycle and negatively impact brain health.^99^ In sum, modern social factors often misalign with adequate sleep. Tackling sleep disparities requires addressing the social exposome and its impacts on brain health. In general, exposure to physical and social changes in our environment plays a critical role in shaping the sleep health of inhabitants of the planet. Interestingly, poor sleep health can also intensify social challenges by worsening mood, cognition, emotion regulation, well-being, and interpersonal relationships in families, schools, and workplaces.^4^^,^^45^ These changes promote further unhealthy lifestyle choices, such as higher alcohol intake or smoking, poor diet, physical inactivity, which together contribute to a feedback loop linking human health with the cumulative exposome (Figure 1B).

## Recommendations for improving global sleep health

Elevating sleep health to a global priority will require concerted action on transdisciplinary fronts. The lack of international coordination impedes the sharing of best practices and the implementation of coordinated interventions across countries. Recently, we have advocated a comprehensive *sleep diplomacy* approach,^20^ analogous to climate or vaccine diplomacy. Nations should collaborate interactively to promote OSH as a fundamental component of well-being for humans and animals alike. Coordinated international *sleep diplomacy* includes global partnerships, summits (e.g., the World Sleep Health Summit), policy harmonization, and shared resources to support countries with limited infrastructure in promoting sleep health as a global priority. Policies could introduce new scales, such as a *Sleep Health Impact Index*, to assess the impact of new regulations on community sleep. These should be standardized population-level tools that quantify how health, lifestyles, urban planning, the environment, labor, education, and digital policy affect sleep health in society. Multidimensional sleep outcomes (duration, timing, regularity, efficiency, and sleep disorder burden) may serve as upstream exposome indicators (e.g., light at night, noise, air pollution, work schedules, school start times, housing density, and digital environments), weighted by age, sex, socioeconomic status, and occupational risk. Importantly, these indices could be applied both prospectively and retrospectively to assess how policies (nighttime lighting regulations, shift-work legislation, urban greening, and school scheduling) translate into measurable changes in population sleep health. These indices would serve as accountability tools, analogous to health or environmental impact assessments, embedding sleep health within public health and governance within the OSH framework. As with climate or vaccine diplomacy, global sleep advocacy can yield substantial returns in cognitive health, productivity, and resilience. Research findings need active engagement with policymakers and other stakeholders to influence real-world decisions.^100^ Subsequently, we outline key strategies and recommendations to advance global sleep health policy (Box 1).

• ***At the individual level*,** public campaigns to increase awareness and provide sleep hygiene education are essential to induce behavioral change and boost sleep literacy across all age groups, particularly among adolescents. These should promote practical sleep hygiene tips through culturally tailored messaging in schools, workplaces, podcasts, and social media. Medical, nursing, and veterinary curricula should also integrate training in sleep science and chronobiology to address critical gaps in clinical and animal care.
• ***At the clinical level*,** interventions such as tailored light exposure, cognitive-behavioral therapy for insomnia (CBT-I), continuous positive airway pressure for OSA, pharmacotherapy (when necessary), and aligning sleep schedules closer to personalized natural circadian rhythms can improve sleep health, mood, emotional processing, cognitive performance, and cardiometabolic systems.^30^^,^^50^
• ***At the research level*,** critical steps are required, as most countries lack population-level sleep data. We call for standardized data collection, open-access global sleep databases, and interdisciplinary research linking sleep with the exposome, mental health, aging, and neurobiology. Integrating sleep outcomes into existing longitudinal cohorts and trials would enhance public health surveillance with minimal added cost. Inter-individual variability and cultural and geographical differences in sleep patterns and their health impact are often overlooked,^21^^,^^23^ and can be captured by precision sleep medicine approaches. Precision medicine requires neuroimaging, biomarkers, artificial intelligence, whole-body health, and exposome measures^12^ to develop personalized interventions that enhance sleep health. Future research should focus on utilizing AI-driven personalized monitoring, interventions, and treatments for sleep disorders. For example, applying personalized approaches to optimize sleep at an individual level is the next frontier for the ENIGMA-Sleep consortium (https://enigma.ini.usc.edu/ongoing/enigma-sleep/). It involves pooling brain imaging, behavioral, and genomic data across thousands of individuals from multiple international sites to identify neurobiological biomarkers of sleep disturbances and novel therapeutic targets. In parallel, novel consumer sleep technologies (wearable sleep trackers, home electroencephalogram (EEG), remote sensing of environmental conditions, and app-based measurements) and big data analytics using AI algorithms enable longitudinal, real-life monitoring and feedback, opening the door to *sleep health coaching* tailored to one’s lifestyle and environment. Combined with traditional public health measures, these innovations could reduce the global burden of sleep-related chronic diseases.
• ***At the public health level*,** *sleep capital* remains an underdeveloped economic asset.^19^ Insufficient sleep incurs massive economic costs (through reduced productivity, motor vehicle accidents, and healthcare expenses). However, unlike nutrition or vaccination, sleep has received relatively little investment in prevention and education campaigns, particularly for children and adolescents. Urban planning that considers circadian health (e.g., reducing light, noise, and air pollution, enforcing quiet nighttime hours, and increasing green spaces) could benefit both people and urban wildlife. In workplaces and schools, aligning schedules more closely with humans’ biological clocks (e.g., later school start times for adolescents, or strategic shift scheduling for workers) can improve school and job performance and safety. Integrating chronobiology principles into public health and even veterinary care (e.g., timing medical treatments to an organism’s circadian phase) could yield better outcomes across the board. In addition to healthy nutrition and physical activity, governments and international health agencies should formally incorporate sleep health into their health policies. For example, they should create dedicated regional units, integrate sleep metrics into national surveys, and set global targets, such as reducing the prevalence of poor sleep health by 2035. Urban planning, workplace regulations, and school curricula should align with circadian principles to promote environments conducive to healthy sleep. The concept of sleep capital aligns closely with the OSH model by framing sleep health as a foundational driver of socio-economic development and arguing that strategic investments in it can yield substantial returns in productivity, mental well-being, and overall societal resilience.

## Outlook

The world must wake up to the silent epidemic of poor sleep health. Our proposed OSH framework emphasizes the complex interconnections among human, animal, and environmental health, which influence ecosystems, biodiversity, and overall well-being. The sleep health must be elevated as a global public health imperative. The challenges to healthy sleep, from climate change to unhealthy lifestyles and social factors, are complex and transboundary, requiring a multifaceted response. This framework can help to unify efforts in health, medicine, psychology, neuroscience, veterinary science, and environmental policy to address the root causes of sleep disturbances. Investing in education, research, and international development can build the sleep capital for societies, reaping benefits in well-being, economic productivity, and resilience.

## Acknowledgments

Sarah Genon is supported by the Deutsche Forschungsgemeinschaft (DFG, GE 2835/2–1, GE 2835/9-1). Agustin Ibanez is supported by grants from the Multi-partner consortium to expand dementia research in Latin America (ReDLat2, supported by Fogarty International Center [FIC], National Institutes of Health, National Institutes of Aging [R01 AG057234, R01 AG075775, R01 AG21051, R01 AG083799, CARDS-NIH, R01 AG057234], Alzheimer's Association [SG-20-725707], Rainwater Charitable Foundation – The Bluefield project to cure FTD, and Global Brain Health Institute), ANID/FONDECYT Regular (1250091 and 1210176 and 1220995); ANID/PIA/ANILLOS ACT210096; JPI JPND-Care, DISCeRN 2025 - Health and Social Care Research with a Focus on the Moderate and Late Stages of Neurodegenerative Diseases; FONDEF ID20I10152, and ANID/FONDAP 15150012; Wellcome Trust award for BRAIN-CLIMA: Investigating the Combined Impact of Heat and Air Pollution on Blood-Brain Barrier Integrity and Brain Aging in Latin America, (335293/Z/25/Z), Wellcome Leap CARE Program (grant no.: CARE-2025-0883490149) for the project “Advancing Female-Specific Predictive Models and Risk Assessment Tools for Alzheimer’s Disease in the US and Latin America, and the CliCBrain (Horizon ID: 101236426; https://doi.org/10.3030/101236426, Marie Skłodowska-Curie Actions - MSCA). The contents of this publication are solely the responsibility of the authors and do not represent the official views of these institutions. The funders had no role in study design, data collection and analysis, decision to publish, or preparation of the manuscript. The contents of this publication are solely the author’s responsibility and do not represent the official views of these institutions.

## Declaration of interests

The authors declare no competing interests.

## Supplemental information

Document S1. Box S1

Document S2. Article plus supplemental information
