# Supplementary material for: Elevating sleep to a global health priority: The One Sleep Health framework
Source: Cell Rep Med. 2026 May 22;7(6):102828. doi: 10.1016/j.xcrm.2026.102828 (PMC13293962; doi:10.1016/j.xcrm.2026.102828)
Supplement: Document S1. Box S1 [file mmc1.pdf]

Cell Reports Medicine, Volume *7*

Supplemental information

Elevating sleep to a global health priority:

The One Sleep Health framework

Masoud Tahmasian, Vincent Küppers, Sarah Genon, Simon B. Eickhoff, Diego A. Golombek, and Agustin Ibanez

Supplementary file

Tahmasian et al. Elevating Sleep to a Global Health Priority: The One Sleep Health Framework

Box S1. Definition of key terms for the OSH framework

One Sleep Health: An integrated, transdisciplinary framework, linking human sleep health, animal sleep health, and the physical/social/lifestyle exposome.

Sleep Capital: The cumulative benefits of high-quality sleep, including its socio-economic impacts. This underscores the importance of sleep as an economic asset, advocating for investments in sleep health to enhance productivity, mental well-being, and societal resilience.

Sleep Diplomacy: The strategic promotion of sleep health on an international scale. This involves policy interventions, advocacy, and international collaborations to address worldwide sleep health disparities and make it a global priority.
